# Bi-Level Simulation-Driven Optimization for Route Guidance in Disrupted Metro Networks via Hybrid Swarm Intelligence

**DOI:** 10.3390/s26051711

**Published:** 2026-03-08

**Authors:** Xuanchuan Zheng, Yong Qin, Jianyuan Guo, Xuan Sun, Guofei Gao

**Affiliations:** 1 School of Traffic and Transportation, Beijing Jiaotong University, Beijing 100044, China; 20114089@bjtu.edu.cn (X.Z.); xuansun@bjtu.edu.cn (X.S.); 2 State Key Laboratory of Advanced Rail Autonomous Operation, Beijing Jiaotong University, Beijing 100044, China; 3 CRSC Research & Design Institute Group Co., Ltd., Beijing 100070, China; 4 National Engineering Research Center of Urban Rail Transit Green and Safe Construction Technology, Beijing Urban Construction Design & Development Group Co., Ltd., Beijing 100037, China; gaoguofei@bjucd.com

**Keywords:** urban rail transit, route guidance, simulation-driven optimization, hybrid swarm intelligence, adaptive large neighborhood search (ALNS)

## Abstract

Real-time route guidance during disruptions in urban rail transit systems requires rapidly providing effective strategies that simultaneously alleviate congestion and account for passengers’ travel time. This study proposes an optimization framework that considers travel time, congestion perception time, and information costs, incorporating a Logit choice model with information bias to reflect passengers’ behavioral responses under disruptions. A bi-level simulation evaluation mechanism is employed to rapidly evaluate the objective functions under different guidance strategies, where a Physically Consistent Incremental Simulator, based on differential computation, achieves a 599-fold speedup while maintaining high fidelity with full-scale simulations (Pearson correlation > 0.96). A hybrid algorithm combining the Gray Wolf Optimizer and Adaptive Large Neighborhood Search is developed to solve the origin–destination level route guidance optimization problem. The algorithm embeds domain knowledge-based “destroy and repair” operators with a sequential repair mechanism to enable fast global search and precise local refinement. Case study results demonstrate that the framework reduces severely congested sections by 36%, shortens average travel time by 7.16 min, and improves solution quality by 12–30% over baseline algorithms. These findings confirm the practical applicability of integrating intelligent optimization with high-efficiency simulation for emergency route guidance in large-scale metro networks.

## 1. Introduction

As the backbone of urban mobility, the increasing complexity of metro systems inherently introduces significant vulnerability. With rapid network expansion and infrastructure aging, unplanned service disruptions—ranging from signal failures to rolling stock malfunctions—have become frequent. Unlike minor delays, these stochastic incidents trigger immediate spatiotemporal backlogs and cascading congestion, resulting in an exponential rise in system disorder and posing a threat to operational safety [1].

In the context of Smart Urban Rail, traditional static emergency plans focusing on supply-side adjustments (e.g., train rescheduling) are insufficient to manage such non-linear dynamics. The system demands Autonomous Resilience—the intrinsic capability to self-organize and restore equilibrium. Consequently, route guidance has emerged as a critical mechanism. By proactively reshaping demand-side distribution through information intervention, route guidance aligns passenger flow with degraded supply constraints [2]. However, realizing high-precision autonomous control faces three fundamental obstacles:

Comprehensive Optimization: Decisions must navigate the trade-off between minimizing system delays (Efficiency) and mitigating psychological panic caused by overcrowding (Experience), a balance often missed by single-objective models.

Computational Bottleneck: Reconfiguring paths for thousands of OD pairs in real-time is an NP-Hard problem. Traditional full-scale simulation is too slow to support decision-making within the “golden window” of emergency response.

Decision Precision: Existing heuristics struggle to balance global exploration with local exploitation, often yielding generic schemes that lack the granularity to resolve specific bottlenecks.

Bridging the gap between high-fidelity simulation and real-time optimization constitutes the primary motivation of this work. To address these challenges, this paper proposes a simulation-driven intelligent control framework integrating digital twin technology with swarm intelligence.

### 1.1. Literature Review

To address the critical challenges identified above, this section reviews the state of the art across three dimensions: comprehensive optimization models, passenger flow evolution inference, and intelligent solution algorithms.

#### 1.1.1. Comprehensive Optimization and Passenger Behavioral Modeling

Constructing a scientific optimization model is the core of passenger flow control. The evolutionary path has shifted from a singular focus on system efficiency to a mu lti-dimensional balance involving equity and behavioral complexity. Early foundational models [3] predominantly aimed at System Optimum (SO) by minimizing total network delay. However, Tirachini et al. (2013) [4] argue that disregarding the negative utility of crowding leads to overestimating service levels. Consequently, recent studies have pivoted towards comprehensive optimization frameworks. For instance, Cadarso et al. [5] develop bi-level programming models to trade off operational costs against passenger delay. More recently, Zhou et al. (2025) [6] extend this to include low-carbon objectives, reflecting the growing complexity of operational goals.

Crucially, the fidelity of these models relies on the granular characterization of passenger behavior. Unlike the perfect rationality assumed in traditional Logit models [7], decision-making under disruptions implies a binary game with limited information. Di & Liu (2016) [8] introduce the Bounded Rationality theory to define decision boundaries under uncertainty. Furthermore, Kattan et al. [9] and Ben-Elia & Avineri [10] emphasize Information Compliance Bias, confirming that ignoring passengers’ intrinsic trust in guidance significantly undermines model robustness. Current research, however, often lacks a unified framework that simultaneously quantifies the non-linear “psychological penalty” of crowding and the stochastic “compliance behavior” of passengers in real-time.

#### 1.1.2. Data-Driven and Simulation-Based Methodologies

Accurately inferring the spatiotemporal distribution of passenger flow is a prerequisite for effective control. Methodologies generally fall into data-driven approaches and Multi-Agent Simulation (MAS). Data-driven methods utilizing smart card data [11] or deep learning have evolved significantly. For example, Li et al. (2024) [12] utilize complex network theory and the Informer model to capture non-linear dependencies in rail flows. He et al. [13] propose a deep reinforcement learning (DRL) framework integrating graph representation and self-attention mechanisms to dynamically generate passenger route guidance strategies in multimodal transit systems. While DRL and deep learning models offer rapid online inference, these methods often suffer from poor generalization due to the sparsity of historical data on severe, zero-shot disruptions [14].

Conversely, MAS has become the mainstream choice for characterizing emergent phenomena in complex adaptive systems. Li & Zhu [15] and Yin et al. [16] utilize MAS to reproduce station closures and dynamic path reconfiguration. Recent advancements focus on Digital Twin technologies to bridge the gap between simulation and reality [17]. However, a critical gap remains: the prohibitive time cost of full-scale microscopic simulation (often hours) fails to meet the “golden window” requirement (typically minutes) for emergency response [18], creating an urgent need for efficient incremental evaluation mechanisms that retain physical realism while enabling high-frequency iterations.

#### 1.1.3. Metaheuristics and Hybrid Optimization Algorithms

The route guidance problem represents a high-dimensional, dynamic, NP-Hard combinatorial optimization problem. Classic metaheuristics like Differential Evolution (DE) [19], Whale Optimization Algorithm (WOA) [20], and Gray Wolf Optimizer (GWO) [21] are widely used for their global search capabilities. However, when applied to discrete 0–1 decision spaces in large-scale networks, these algorithms frequently suffer from “premature convergence.” To address this, recent studies have developed various GWO variants, such as incorporating dynamic convergence factors, orthogonal learning strategies, or discrete mapping mechanisms, to enhance global exploration capabilities in complex routing and scheduling optimization tasks [22,23].

To mitigate this, Adaptive Large Neighborhood Search (ALNS) [24] allows for structured exploration through dynamic “destroy” and “repair” operators. The adaptability of ALNS in highly stochastic and dynamic environments has been well documented; for instance, Liu et al. [25] successfully apply an ALNS-based approach to dynamically solve the meal delivery routing problem with a hybrid fleet of riders and autonomous vehicles, demonstrating its superior capability in handling complex network reconfigurations. Recent hybrid variants have demonstrated considerable potential in improving solution quality. However, these approaches often lack domain-specific mechanisms, such as bottleneck removal strategies tailored to metro networks, which limits their precision in mitigating complex congestion propagation [26].

In summary, while significant progress has been made, there remains a lack of an integrated framework that can simultaneously address the behavioral uncertainty of passengers, the real-time computational constraints of simulation, and the precision of high-dimensional optimization. This study aims to bridge these gaps.

### 1.2. Contributions and Structure

To address the challenges of real-time passenger flow control under sudden disruptions, this study makes three contributions.

(1) A Comprehensive Optimization Framework: We construct a dynamic model balancing system efficiency, congestion perception, and intervention scale. By integrating a Logit model that accounts for “Information Compliance Bias,” the framework accurately captures passenger bounded rationality, transforming theoretical flow control into realistic, behavior-aware guidance.

(2) A Physically Consistent Incremental Simulator ($F_{1}$): We innovatively design a surrogate engine based on differential calculation technology to reconcile evaluation fidelity with computational speed. Integrated within a bi-level architecture alongside the full-scale simulator ($F_{2}$), this mechanism enables high-frequency iterative optimization while strictly adhering to physical network constraints.

(3) A Hybrid Swarm Intelligence Algorithm (GWO-ALNS): We propose a decision-making engine that synergizes the global exploration of GWO with the local refinement of ALNS. Featuring innovative goal-oriented “destroy and repair” operators, this hybrid algorithm drives deep iterative optimization, ensuring precise solution discovery within the high-dimensional discrete decision space.

The remainder of this paper is organized as follows. Section 2 establishes the problem formulation and route reconstruction logic. Section 3 details the comprehensive Optimization model. Section 4 presents the hybrid GWO-ALNS algorithm. Section 5 provides a case study validation on the Chongqing Rail Transit network. Finally, Section 6 concludes the study.

## 2. Problem Statement

This section formalizes the route guidance problem under disruptions as a dynamic combinatorial optimization process. We define the network physics, decision logic, and computational characteristics to establish the mathematical foundation for the optimization model.

### 2.1. Mathematical Description of the Problem

We model the urban rail transit network as a directed graph G=(N,E), where N denotes the set of stations (nodes) indexed by n, and E denotes the set of track sections (edges) indexed by e. A disruption is defined as a time-dependent capacity constraint. An incident on section $e_{fault}\in E$ during $\left[t_{start},t_{end}\right]$ enforces a residual capacity ${\;C}_{e}(t)$ such that available supply is strictly insufficient to meet the dynamic demand $D_{e}(t)$. This supply-side shock triggers a non-linear state evolution. Unlike static traffic assignment, the network state vector S(t) is path-dependent and governed by queuing dynamics at nodes n∈N. The congestion propagates via a spatiotemporal diffusion process, where local bottlenecks induce global perturbations.

### 2.2. Formulation of the Route Guidance Decision

To bridge the gap between supply and demand, the core task is to reallocate spatiotemporal resources. We formulate this as a decision-making problem concerning path search scope and control variable dimensions.

**Figure 1:**
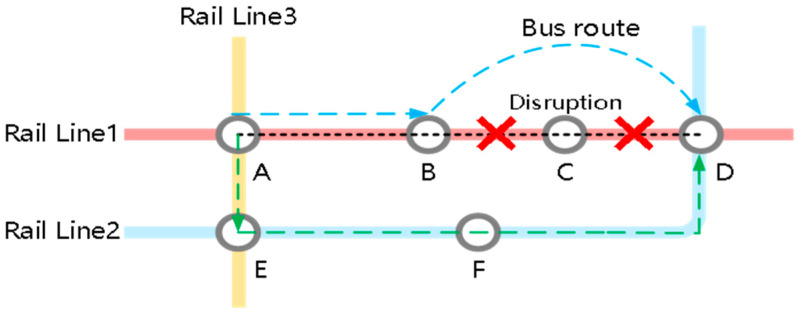
Simple network under sudden operational incidents.

(1) Feasible Path Scope and Reconstruction: The route guidance strategy targets the set of Directly Affected Passengers (DAPs), denoted as K, whose travel is impeded by the topological changes shown in Figure 1. For each affected OD pair k∈K, the feasible path set is reconstructed into a discrete candidate set $R_{k}$ to model a binary decision process (illustrated in Table 1). This set comprises two distinct options: the Original Path (r=0), which implicitly represents the passive strategy of waiting at the platform for service recovery, and the Recommended Path (r=1), representing the active strategy of taking a system-optimal detour generated via shortest path search on the reconstructed topology.

(2) Decision Variables and Dimensions: We define the control variable as a binary vector X. For each affected OD pair k∈K, the decision variable $x_{k}$ determines the intervention strategy: let $x_{k}=1$ denote activating the route recommendation (pushing path r=1), and $x_{k}=0$ denote no intervention (maintaining the original path r=0). Thus, the global decision vector is represented as $X=\{x_{1},x_{2},...,x_{\left|K\right|}\}$, where the dimension of this vector corresponds strictly to the number of affected OD pairs $\left|K\right|$.

### 2.3. Computational Complexity Analysis

Finding the optimal vector X to minimize system cost is a non-trivial engineering challenge, mathematically characterized by three properties:

(1) Combinatorial Explosion (NP-Hard): Since X is a binary vector of length $\left|K\right|$, the solution space X forms a discrete hypercube $X=\{0,1{\}}^{\left|K\right|}$. With $\left|K\right|$ typically reaching $10^{3}∼10^{4}$ in large networks, the search space $2^{\left|K\right|}$ grows exponentially, rendering exact algorithms infeasible.

(2) Coupling of Nonlinearity and Stochasticity: The mapping from the decision vector X to the system objective Z is non-analytical. The crowding penalty function creates a non-convex landscape with multiple local optima, while the Bounded Rationality of passengers (Logit model) introduces stochastic noise into the evaluation.

(3) Black-Box Characteristics: Due to the complex interaction between trains v∈V and passengers, there is no explicit gradient expression ∇Z. The system acts as a “Black Box”, necessitating a derivative-free, simulation-driven metaheuristic approach for solution searching.

### 2.4. Basic Modeling Assumptions

To construct a tractable mathematical model (detailed in Section 3) that captures these core dynamics, the following necessary assumptions are established:

Precise Targeting: Guidance information is pushed exclusively to the DAP set (K). IAP flows are treated as dynamic environmental constraints.

OD Granularity Consistency: The OD pair serves as the atomic decision unit. An “All-or-Nothing” strategy is applied, assuming homogeneity in information reception within the same OD group.

Baseline Behavioral Inertia: In the absence of intervention ($x_{k}=0$), or if guidance is ignored, passengers are assumed to maintain their original path (including waiting at platforms).

Bounded Rationality: Passengers receiving guidance ($x_{k}=1$) do not deterministically obey. Instead, they execute a probabilistic choice based on the utility difference between paths, governed by the Logit model.

Strict Capacity Constraints: Train capacity is strictly enforced. When demand exceeds train capacity $C_{v}$, the simulation enforces a truncation mechanism, where excess passengers constitute a “retention queue” at the station nodes N.

### 2.5. Symbol Definitions

To clearly describe the model construction process, the definitions of the main sets, parameters, and decision variables involved in this paper are shown in Table 2.

## 3. Optimization Model

This section provides a mathematical optimization framework that links macroscopic control strategies with microscopic passenger behavior. It refines decision granularity to the level of individual affected OD pairs to enable high-precision flow control under disruptions. A weighted objective function is constructed with respect to a baseline scenario, aiming to balance system efficiency, passenger experience, and guidance costs while maintaining robust convergence.

### 3.1. Model Framework

**Figure 2:**
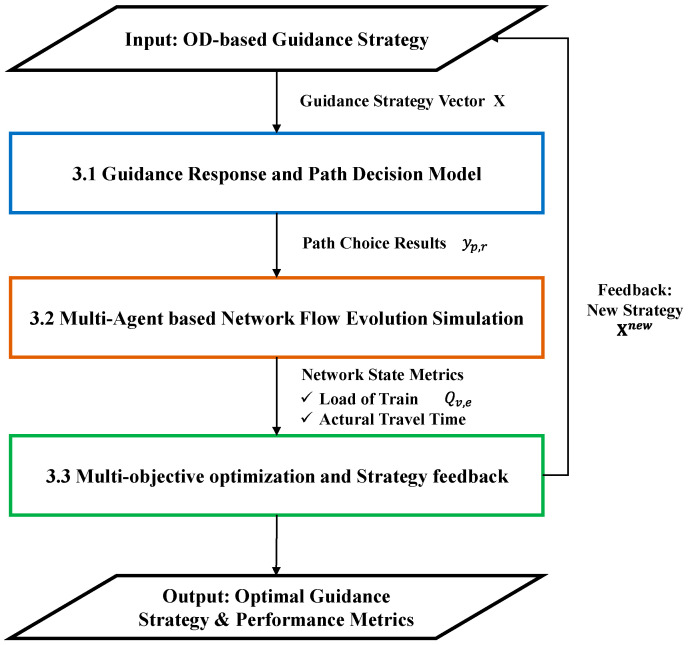
Optimization framework for passenger flow guidance.

As illustrated in Figure 2, the proposed framework adopts a hierarchical closed-loop structure. This architecture facilitates a seamless transition from strategy formulation to performance evaluation and feedback, ensuring that guidance decisions are continuously adjusted according to simulated network conditions. The overall logic is implemented through three tightly integrated functional layers.

The optimization process begins by mapping the macroscopic guidance strategy vector X to individual path choices $y_{p,r}$, which characterize passengers’ detour decisions in response to guidance and provide the behavioral input for network simulation.

Using $y_{p,r}$ as demand input, the network evolution is then simulated in a multi-agent environment, dynamically generating key performance indicators such as train load factors $Q_{v,e}$ and realized travel times that describe the system state under the current strategy.

These indicators are subsequently evaluated against the baseline, and the comprehensive optimization module updates the strategy to $X^{new}$, which is fed back to the behavioral response stage; this closed-loop coupling among behavior, network state, and strategy refinement drives the system toward a near-global optimum that balances efficiency and travel quality.

### 3.2. Guidance Response and Path Decision Model

As the core of the lower-level multi-agent simulation (MAS), the guidance response model characterizes the mapping process from macroscopic OD-level strategies to microscopic individual path choices. It describes the behavioral mechanism by which system-level information is translated into discrete decisions by passengers.

#### 3.2.1. Generalized Travel Cost Construction

Considering passenger sensitivity to crowding during disruptions, a generalized cost function is constructed. It integrates travel time, transfer penalties, and non-linear crowding perception. For passenger p in OD pair k choosing path r, the generalized cost $C_{p,r}$ is defined as follows:

(1) $$C_{p,r}=t_{p,r}^{wait}+t_{p,r}^{walk}+t_{p,r}^{trans}+{\beta_{trans}\cdot N}_{p,r}^{trans}+\sum_{e\in r}\left(t_{v,e}\cdot \left(1+\alpha_{crowd}\cdot \rho_{v,e}\right)\right)$$

where $t_{p,r}^{wait},\;t_{p,r}^{walk},$ and $t_{p,r}^{trans}$ represent waiting, access/egress walking, and transfer walking time, respectively;$\cdot N_{p,r}^{trans}$ represents the number of transfers, $\beta_{trans}$ is the psychological impedance penalty coefficient per transfer [23]; the final term reflects the amplification effect of standing density $\rho_{v,e}$ on time perception [27].

#### 3.2.2. Logit Choice Model with Information Bias

Addressing the bounded rationality under emergency scenarios, we assume that informed passengers engage in a binary choice between the original path (r=0) and the recommended path (r=1). The probability $P_{p,r}$ of passenger p choosing the recommended path is governed by a Logit model:

Addressing bounded rationality, informed passengers engage in a binary choice between the original path (r=0) and the recommended path (r=1). The probability $P_{p,r}$ is governed by a Logit model, with the utility function $V_{p,r}$ integrated as follows:

(2) $$V_{p,r}=-\theta \cdot C_{p,1}+r\cdot \theta \cdot B_{info},r\in \{0,1\}$$

(3) $$P_{p,r}=\frac{\exp(V_{p,r})}{\exp(V_{p,r=0})+\exp(V_{p,r=1})}$$

where θ is the sensitivity coefficient [28], and $B_{info}$ is the Information Compliance Bias [9,10,29]. This bias characterizes the intrinsic trust in system recommendations; when r=1, $B_{info}$ provides a utility gain that may lead passengers to choose a recommended path even if its physical cost is slightly higher.

#### 3.2.3. Decision Implementation via Bernoulli Sampling

To bridge these probabilistic choices with the microscopic simulation, the final path choice result $y_{p,r}$ is determined. For an informed OD pair ($x_{k}$ = 1), the individual decision is realized via Bernoulli Sampling:

(4) $$y_{p,r}∼Bernoulli(P_{p,r})$$

where $y_{p,r}$ = 1 indicates the passenger accepts the guidance and switches to the detour path. This discrete selection $y_{p,r}$ serves as the direct input for the Network Evolution Layer (Module 3.2), driving the subsequent spatiotemporal flow distribution.

### 3.3. Two-Level Simulation Evaluation Mechanism

**Figure 3:**
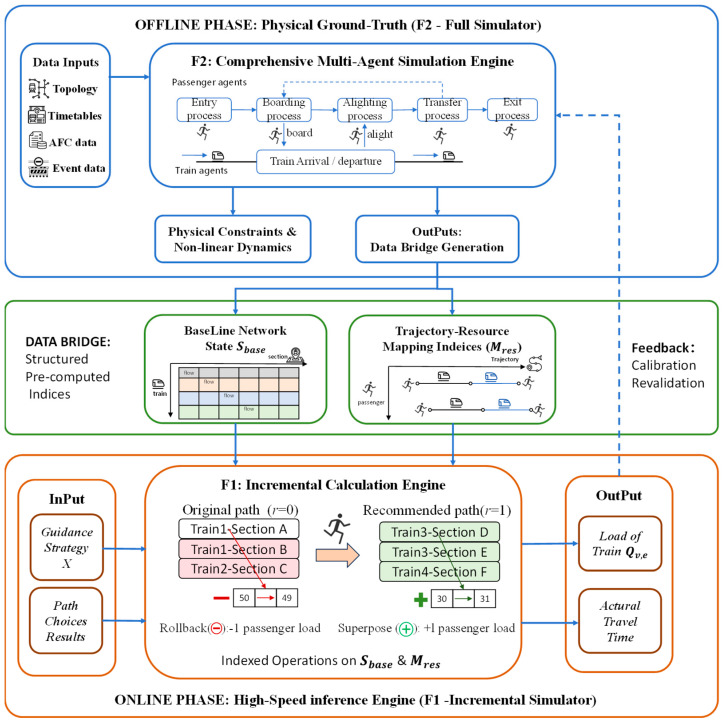
Two-level simulation evaluation framework.

To overcome the high computational demands of microscopic multi-agent simulation (MAS), this paper proposes a double-layer collaborative evaluation mechanism. As depicted in Figure 3, this mechanism employs a “Master–Slave” relationship between the full-scale simulator $F_{2}$ and the incremental simulator $F_{1}$ to balance physical fidelity with computational tractability.

#### 3.3.1. System Workflow and Architecture

The framework executes a structured transition from global physical modeling to localized incremental updates. The complete workflow consists of four primary stages:

(1) Offline Initialization and $F_{2}$ Execution. $F_{2}$ loads comprehensive multi-source data, including network topology, timetables, and Automatic Fare Collection (AFC) records. It simulates all passenger agents to establish a high-fidelity physical ground truth.

(2) Data Bridge Generation. Results from $F_{2}$ are extracted and converted into structured pre-computed indices. This module solidifies the baseline network state ($S_{base}$) and trajectory–resource mapping indices ($M_{res}$).

(3) Online $F_{1}$ Evaluation. During the optimization loop, the $F_{1}$ engine receives guidance strategy candidates. It leverages the Data Bridge to perform high-speed incremental calculations.

(4) Closed-Loop Calibration. The framework periodically invokes $F_{2}$ to validate $F_{1}$’s inference results. This feedback loop ensures accuracy under complex non-linear constraints.

#### 3.3.2. Core Functional Modules

(1) ${\;F}_{2}$: Physical Ground-Truth Generator and Microscopic Simulator

The $F_{2}$ module serves as the high-fidelity foundation of the evaluation framework by executing full-scale microscopic simulations for all passenger and train agents. It explicitly models the complete spatiotemporal lifecycle of every individual passenger, including the entry process, boarding and alighting dynamics, complex transfer maneuvers, and final exit procedures [15,16,18]. During these processes, $F_{2}$ accounts for intricate train-passenger interactions and non-linear physical constraints, such as boarding competition on station platforms and train residual capacity effects [30,31]. Because the simulator must process full-volume AFC records and train timetables while adhering to these high-fidelity behavioral rules, it inherently incurs a massive computational burden. Ultimately, $F_{2}$ establishes the “physical benchmark” by identifying Directly Affected Passengers (DAP) and generating a precise snapshot of the baseline network state.

(2) Data Bridge: Structured Pre-Computed Indices

The Data Bridge enables seamless transition from $F_{2}$’s computationally intensive outputs to $F_{1}$’s rapid inference by generating structured indices. It solidifies baseline flow snapshots ($S_{base}$) and passenger-specific trajectory–resource mappings ($M_{res}$) for all affected passengers. By pre-computing resource occupation sequences linking individuals to line–direction–train–section units, this layer creates a static data infrastructure for online evaluation—effectively pre-computing the global network state to let the optimization engine focus solely on local flow perturbations, eliminating redundant recalculations.

(3) $F_{1}$: High-Speed Incremental Engine and Differential Logic

Operating as a high-speed inference engine, $F_{1}$ exploits the sparse perturbation nature of guidance strategies for rapid fitness evaluations. Rather than full network re-simulation, $F_{1}$ applies “Rollback & Superposition” via Data Bridge indices, modeling state evolution as follows:

(5) $$S_{new}=S_{base}⊖L\left(\Omega_{switch}^{old}\right)⊕L\left(\Omega_{switch}^{new}\right)$$

In this formulation, original path resource loads are stripped (⊖) from the baseline, while newly recommended path loads are superposed (⊕). Supported by bi-directional mapping mechanisms ($M_{A\to R}$) and ($M_{R\to A}$), $F_{1}$ reduces computational complexity from global network scale to local perturbation scale.

This dimensionality reduction enables thousands of optimization iterations within strict time constraints, confining state updates to affected local topology subsets and eliminating redundant calculations for unaffected regions.

### 3.4. Comprehensive Optimization Function

#### 3.4.1. Objective Function

To eliminate differences in dimensions (travel time, perceived crowding time, and message cost) and prevent the optimization process from being dominated by objectives with large numerical values, a normalized objective function based on the change rate from the baseline scenario is constructed. The total objective function Z is defined as the weighted sum of three dimensionless sub-objectives:

(6) $$\min\;Z\;=\;w_{1}\cdot F_{time}\;+{\;w}_{2}\cdot F_{crowd}\;+{\;w}_{3}\cdot F_{msg}$$

The definition and calculation of each component are as follows:

(1) Normalized Physical Travel Time Change Rate ($F_{time}$)

This term measures the physical efficiency improvement brought by the guidance strategy. It tallies only the actual physical time consumed (including waiting, riding, and walking) and transfer penalties, excluding the crowding perception multiplier to maintain the purity of the physical quantity and avoid double-counting with $F_{time}$.

(7) $$F_{time}=(Z_{time}\left(X\right)-Z_{time}^{0})/Z_{time}^{0}$$

where the Total Travel Time under the current scheme X is calculated as follows:

(8) $$Z_{time}\left(X\right)\;=\;\sum_{k\in K}\sum_{p\in P_{k}}\sum_{r\in R_{k}}y_{p,r}\cdot \left(t_{p,r}^{wait}\;+\;t_{p,r}^{walk}\;+{\;t}_{p,r}^{trans\;}\;+\sum_{e\in r}t_{v,e}\right)$$

Physical Meaning: $F_{time}$ is typically negative. For example, $F_{time}$ = −0.05 indicates that the total physical travel time of affected passengers is reduced by 5% compared to the baseline scenario without guidance.

(2) Normalized Crowding Perception Penalty Improvement Rate ($F_{crowd}$)

This term measures the improvement in passenger experience and safety. It quantifies only the Extra Psychological Penalty caused by excessive crowding (e.g., high standing density).

(9) $$F_{crowd}=(Z_{crowd}\left(Q\right)-Z_{crowd}^{0})/Z_{crowd}^{0}$$

where the Total Crowding Penalty under the current scheme is calculated based on section flow $Q_{v,e}$:

(10) $$Z_{crowd}\left(Q\right)\;=\;\sum_{v\in V}\sum_{e\in E_{v}}Q_{v,e}\cdot t_{v,e}\cdot [\alpha_{crowd}\cdot (\rho_{v,e}-\rho_{min})]$$

This formula implies that for passengers on a crowded section e, each person bears an extra psychological penalty time. $\rho_{min}$ represents the crowding density threshold perceived by passengers.

Physical Meaning: A negative $Z_{crowd}\left(Q\right)$ indicates congestion relief. This term guides the algorithm to automatically avoid guidance schemes that cause severe train overloading, preventing “secondary congestion.”

(3) Information Coverage Rate ($F_{msg}$)

This aims to measure the implementation scale of the guidance. It is defined as the ratio of passengers receiving guidance information to the total number of affected passengers:

(11) $$F_{msg}\;=\;[\sum_{k\in K}\left(N_{k}\cdot P_{k}\right)]/N_{total}$$

Physical Meaning: When the improvement effects of $F_{time}$ and $F_{crowd}$ are similar, the model tends to select the scheme with smaller $F_{msg}$, i.e., achieving the same system optimization effect by disturbing fewer passengers.

#### 3.4.2. Constraints

The following constraints define the relationship between passenger flow, train load, and in-train density:

(1) Passenger Load Aggregation

The total passenger load $Q_{v,e}$ of train v on section e is the sum of all passengers assigned to this train that traverse that specific section:

(12) $$Q_{v,e}=\sum_{k\in K}\sum_{p\in P_{k}}\sum_{r\in R_{k}}y_{p,r}\cdot \delta_{p,r}^{v,e},\;\;\forall v\in V,\;\;\forall e\in E_{v}$$

where $\delta_{p,r}^{v,e}$ is a binary indicator variable that equals 1 if passenger p using path choice r takes train v on section e, and 0 otherwise.

(2) Flow Conservation

For train v, the passenger loads on two consecutive sections e−1 and e at station n must satisfy the following flow conservation constraint:

(13) $$Q_{v,e}=Q_{v,e-1}+B_{v,n}-A_{v,n},\;\;\forall v\in V,\;\;\forall n\in N$$

where $B_{v,n}$ and $A_{v,n}$ represent the number of passengers boarding and alighting train v at station n, respectively.

(3) In-Train Standing Density Conversion

The standing passenger density ρ(v,e) is calculated based on the excess load relative to the seating capacity:

(14) $$\rho (v,e)=\max(0,Q_{v,e}-P_{seat,v})/S_{stand,v}$$

where $P_{seat,v}$ represents the total seating capacity (number of seats) of train v, and $S_{stand,v}$ represents the total effective standing area available in train v (m^2^).

(4) Passenger Load Factor

The load factor $\eta_{v,e}$ represents the utilization rate and congestion level of the train:

(15) $$\eta_{v,e}=\frac{Q_{v,e}}{P_{seat,v}+\rho_{std}\cdot S_{stand,v}}\times 100\%$$

where $\rho_{std}$ is the standard standing density (e.g., 6 passengers/m^2^ [32]).

(5) Train Load Factor Constraint

The actual passenger load factor $\eta_{v,e}$ for any train v on any section e must not exceed the maximum allowable threshold $\eta_{max}$.

(16) $$\eta_{v,e\;}\leq {\;\eta }_{max},\;\forall v\in V,\;\forall e\in E_{v}$$

## 4. Hybrid GWO-ALNS Algorithm

### 4.1. Algorithm Framework and Execution Procedure

**Figure 4:**
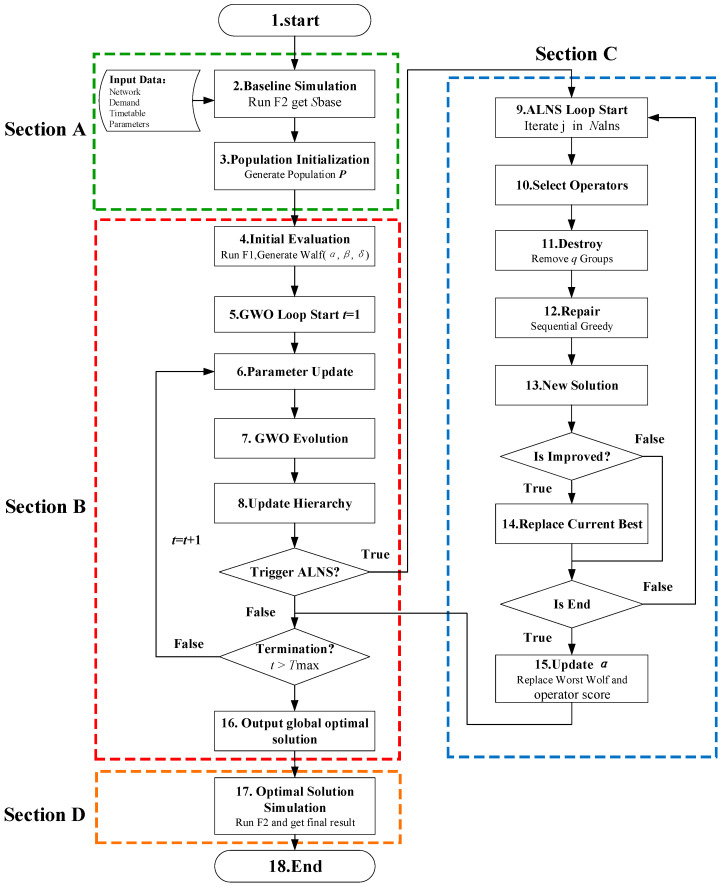
Algorithm overall framework diagram.

To efficiently solve the optimization model defined in Section 3, we propose a hybrid GWO-ALNS algorithm. From an algorithmic perspective, this framework is designed as a hybrid metaheuristic that synergizes the global exploration capabilities of Swarm Intelligence (GWO) with the domain-specific local exploitation strengths of ALNS. As illustrated in Figure 4, the overall framework addresses the NP-hard nature of the route guidance problem by integrating swarm intelligence with domain-specific local search.

The algorithm takes the metro network topology, origin–destination travel demand, and a given disruption scenario as inputs, and determines an optimal guidance strategy specifying whether route guidance should be applied to each OD group.

As shown in Figure 4, the execution procedure is organized into three logical phases (Sections A, B, C, and D), which are described in detail as follows.

#### 4.1.1. Section A: Benchmarking and Initialization

This phase corresponds to the upper block of Figure 4. It establishes the physical baseline and initializes the solution space.

Step 2: Baseline Simulation

The full-scale simulator $F_{2}$ is used to simulate the no-guidance scenario (as illustrated in Figure 3), establishing baseline Total Travel Time $\left(Z_{time}^{0}\right)$ and Congestion Cost $\left(Z_{crowd}^{0}\right)$ for subsequent fitness evaluation.

Step 3: Population Initialization

An initial population of candidate guidance strategies is randomly generated, with each solution encoded as a binary vector indicating active or inactive guidance. Candidates are evaluated using the incremental simulator $F_{1}$, which computes marginal penalty costs without re-simulating the entire network.

#### 4.1.2. Section B: Global Exploration via GWO

This phase, corresponding to the central iterative loop (Section B in Figure 4), employs the Binary Gray Wolf Optimizer (BGWO) [21] as the primary search engine to navigate the high-dimensional solution space.

Step 4: Initial Evaluation.

Upon receiving the initial population P from Section A, the algorithm executes an initial fitness assessment using the incremental simulator $F_{1}$. Based on the fitness values, the social hierarchy is established by identifying the top three leaders: α (Best), β (Second Best), and δ (Third Best).

Step 5 & 6: Loop Start and Parameter Update.

The main optimization loop begins at Step 5 (t=1). In Step 6, the convergence parameter a is updated linearly from 2 to 0 via the formula $a=2-2(t/T_{max})$ [21]. This mechanism enforces a dynamic transition from global exploration (high randomness) in early stages to local exploitation (convergence) in later stages.

Step 7: GWO Evolution.

The remaining wolves (ω) update their positions based on the leaders (α, β, δ). Since the standard GWO operates in a continuous search space, the updated position is first calculated as a continuous vector, denoted as $X_{i}^{c}\left(t\right)=\left[x_{i,1}^{c},x_{i,2}^{c},\ldots ,x_{i,D}^{c}\right]$. To map this continuous estimate to the binary decision space {0,1}, a Shifted Sigmoid Transfer Function $S\left(\cdot \right)$ is applied to each continuous component $x_{i,j}^{c}$:

(17) $$S(x)=\frac{1}{1+e^{-10(x-0.5)}}$$

The final binary decision variable $x_{i,j}\left(t+1\right)$ is then determined stochastically:

(18) $$x_{i,j}\left(t+1\right)=\left\{\begin{array}{ll} 1 & \mathrm{if}\;rand<S\left(x_{i,j}^{c}\left(t+1\right)\right)\\ 0 & \mathrm{otherwise} \end{array}\right.$$

where rand is a uniform random number in [0, 1]. This transformation ensures that the updated decision variables remain feasible binary values.

Step 8: Update Hierarchy.

The newly evolved population is re-evaluated using $F_{1}$, and the social hierarchy (α,β,δ) is dynamically updated.

#### 4.1.3. Section C: ALNS-Based Local Refinement

This phase corresponds to Section C in Figure 4 and serves as a dedicated local refinement module, activated to enhance solution quality and escape local optima using the ALNS framework.

Steps 9 and 10: ALNS Activation and Adaptive Operator Selection.

When a predefined generation-based activation condition is met (Step 9), the ALNS module is triggered to enhance local search. In Step 10, destroy–repair operator pairs are selected via an adaptive roulette wheel mechanism, with probabilities updated based on their historical contribution to solution improvement [24], progressively favoring more effective operators.

Step 11: Targeted Destroy (Perturbation).

The destroy operator selectively removes decision variables associated with the top-q most congested network segments, yielding a partial solution by relaxing dominant local bottlenecks.

Step 12: Sequential Repair (Optimization).

The repair operator reconstructs the partial solution to re-optimize guidance strategies, while the specific operator design is detailed in Section 4.2.

Step 13: Candidate Solution Generation.

A new candidate solution $S_{new}$ is fully generated (Step 13), representing a potential local optimum within the neighborhood of the original solution.

Steps 14 and 15: Evaluation and Feedback Loop.

The algorithm evaluates the candidate solution $S_{new}$ to complete the adaptive feedback loop. If $S_{new}$ outperforms the current global optimum ($S_{\alpha }$), it replaces the α-wolf (Step 14). The refined solution is then injected into the swarm by replacing the worst-performing individual (Step 15), while the scores of the applied operators are adaptively updated to reflect their effectiveness.

#### 4.1.4. Section D: Final Validation and Termination

This final phase, corresponding to Section D in Figure 4, ensures that the optimized strategy is rigorously validated against the ground-truth physical environment.

Step 16: Global Optimal Extraction.

Upon meeting the termination criteria ($t>T_{max}$), the algorithm extracts the global best solution $S^{*}$ from the hybrid GWO–ALNS optimization process, representing the final set of OD groups to be guided.

Step 17: Ground-Truth Simulation.

The extracted solution $S^{*}$ is fed into the full-scale simulator $F_{2}$ for high-fidelity evaluation. $F_{2}$ captures complex non-linear effects, producing the final performance report—including Total Travel Time, Congestion Cost, and detailed passenger trajectories, which are used for evaluating and analyzing the guidance effect, concluding the algorithm.

### 4.2. Domain Knowledge-Based ALNS Framework

The proposed framework is a domain knowledge-based ALNS that integrates passenger-level travel time and congestion diagnostics into the optimization process, enabling targeted and physically interpretable search decisions.

**Figure 5:**
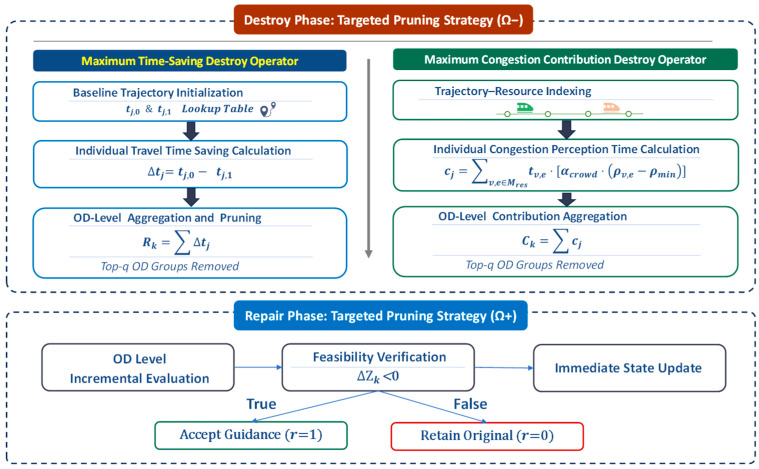
Workflow of the proposed ALNS framework.

Specifically, the framework adopts a targeted prune-and-repair logic: OD groups are first selectively removed based on their quantified contribution to system inefficiency or congestion, and then reinserted through a strictly sequential, feasibility-aware repair process. This design enables effective exploration of the solution space while preserving physical consistency and congestion awareness. The overall workflow of the proposed framework is illustrated in Figure 5.

#### 4.2.1. Mechanism-Driven Destroy Operators $\left(\Omega^{-}\right)$

The destroy phase uses a Targeted Pruning Strategy, with two complementary operators designed to match the two optimization objectives in Section 3.4: one for maximizing travel time savings, and the other for reducing congestion impact. Together, they focus the solver on OD groups with the greatest potential for improvement.

(1) Maximum Time-Saving Destroy Operator

This operator identifies OD groups with the greatest potential for reducing total travel time. It first computes each passenger’s potential travel time savings relative to alternative paths, then aggregates these gains at the OD group level. OD groups are ranked by total savings, and the top-q groups are reset to maximize system-wide travel time reduction.

Step 1: Baseline Trajectory Initialization.

To avoid redundant pathfinding during iterations, we establish a static baseline during the initialization phase. A single execution of the full-scale simulator $F_{2}$ records the travel time for every passenger j under both the original path $\left(t_{j,0}\right)$ and the recommended guidance path $\left(t_{j,1}\right)$. This creates a lookup table for instant retrieval.

Step 2: Individual Travel Time-Saving Calculation.

For each passenger j, the potential net gain from switching strategies is defined as $\Delta t_{j}=t_{j,0}-t_{j,1}$; larger positive values indicate greater individual benefit and thus a stronger preference for recommending a route switch, while negative values imply that switching is unfavorable for the passenger.

Step 3: OD-Level Aggregation and Pruning.

Individual gains are aggregated to compute the Group Regret Score $\left(R_{k}=\sum_{j\in N_{k}}\Delta t_{j}\right)$. OD groups are ranked in descending order of $R_{k}$, and the top-q groups are removed, directing the solver to focus on re-optimizing the most critical efficiency bottlenecks.

(2) Maximum Congestion Contribution Destroy Operator

This operator targets the minimization of total congestion cost $\left(C_{total}\right)$ by identifying the OD groups whose passengers contribute most significantly to network bottlenecks. It efficiently isolates the specific OD flows responsible for critical bottlenecks. Each passenger’s perceived congestion time on overloaded train sections is computed and aggregated at the OD group level to form a Group Congestion Score.

Step 1: Trajectory–Resource Indexing

A Trajectory–Resource Index is precomputed during initialization. This index $M_{res,j}$ maps every passenger j to the specific sequence of train–section resources they occupy along all candidate paths. This structure serves as a high-speed lookup table, allowing the algorithm to instantly identify which passengers are present on any specific train section without global search.

Step 2: Individual Congestion Perception Time

Using the Trajectory–Resource Index and the train–section load table, the algorithm identifies critical links whose crowding exceeds predefined thresholds and retrieves the passengers occupying these sections. Based on Equation (10), each passenger’s congestion perception time is computed as follows:

(19) $$c_{j}=\sum_{v,e\in M_{res,j}}t_{v,e}\cdot [\alpha_{crowd}\cdot (\rho_{v,e}-\rho_{min})]$$

where $t_{v,e}$ is the runtime time of the train on section (v,e), and $\rho_{v,e}$ represents the passenger density inside the train section.

Step 3: OD-Level Contribution Aggregation

Individual costs are summed to the OD group level to derive the Group Congestion Score $\left(C_{k}=\sum_{j\in N_{k}}c_{j}\right)$. OD groups are ranked by $C_{k}$ in descending order, with the top-q groups reset to release capacity at the most critical bottlenecks.

#### 4.2.2. Physics-Aware Sequential Repair Operator $\left(\Omega^{+}\right)$

After the destruction phase, the repair operator reconstructs the solution by reassigning guidance strategies to the unassigned OD groups. To avoid secondary congestion caused by simultaneous re-routing, a strictly sequential repair strategy is adopted. Each unassigned OD group is processed iteratively through a trial–verification–update mechanism:

Step 1: OD-Level Incremental Evaluation

For OD group k, the guidance strategy r=1 is tentatively activated and evaluated using an OD-level incremental evaluation approach (as detailed in Section 4.2.3). Unlike the batch incremental simulation adopted in the GWO phase, which assesses the aggregated impact of multiple OD groups simultaneously, this method isolates the effect of a single group.

The evaluation is conducted on the current network traffic state, which already incorporates flow updates from previously repaired groups. The algorithm computes the marginal change $\Delta Z_{k}$ in the global objective function attributable solely to OD group k, eliminating the need to re-evaluate the entire passenger population.

Step 2: Feasibility Verification

The tentative assignment is evaluated based on $\Delta Z_{k}$. If $\Delta Z_{k}<0$, the guidance strategy is accepted; otherwise, it is rejected and the group reverts to its original path r=0.

Step 3: Immediate State Update

Once the decision for group k is finalized, the network state—particularly the cumulative passenger loads on affected train sections—is immediately updated. This ensures that the capacity consumed by group k is fully reflected when processing the next group (k + 1), enforcing strict sequential dependency and preventing resource conflicts. As a result, the repaired solution remains physically feasible and congestion-aware.

#### 4.2.3. OD-Level Incremental Evaluation and Complexity Analysis

In the ALNS repair phase, each OD group is evaluated sequentially, unlike the batch incremental simulation in the GWO phase that assesses multiple OD groups simultaneously and incurs a computational cost proportional to the number of affected groups. To efficiently determine whether a newly repaired solution improves the global objective function without re-simulating the entire passenger population, an OD-level incremental evaluation strategy is employed.

(1) OD-Level Incremental Evaluation

This evaluation quantifies the marginal impact of a single OD group on the inherited network state $S_{current}$. For a candidate guidance strategy of OD group k with $n_{k}$ passengers, the algorithm superimposes the incremental demand onto the relevant spatiotemporal train–section resources while all other network components remain fixed. The resulting changes in travel time and congestion are denoted as ${\Delta T}_{k}$ and ${\Delta C}_{k}$, respectively. The marginal variation in the global objective function is then computed as $\Delta Z_{k}=w_{1}{\Delta T}_{k}+w_{2}{\Delta C}_{k}$, ensuring that only the contribution of OD group k is captured.

(2) Complexity Analysis

In the GWO phase, evaluating a candidate solution using batch incremental simulation requires aggregating the impacts of all affected passengers, resulting in a computational complexity of $O(N_{all})$. In contrast, the OD-level incremental evaluation in the ALNS phase operates only on the current OD group, yielding a complexity of $O(n_{k}).$ Since $n_{k}\ll N_{all}$, this approach achieves an order-of-magnitude reduction in per-evaluation cost and enables extensive local search within a limited computational budget, corresponding to a theoretical acceleration factor of approximately $N_{all}/n_{k}$.

## 5. Case Study

### 5.1. Data Input and Parameter Settings

#### 5.1.1. Simulation Scenario and Network Data

**Figure 6:**
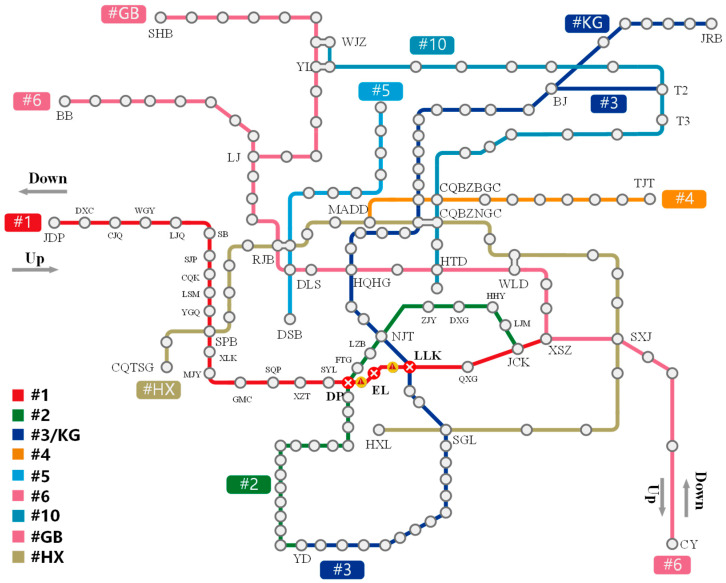
Chongqing rail transit network map under emergency.

To verify the effectiveness of the proposed optimization model, the 2019 Chongqing Rail Transit (CRT) network is selected as the case study (Figure 6), comprising 10 lines and 190 stations. A sudden disruption is configured between Daping (DP) and Lianglukou (LLK) stations on Line 1 during the morning peak (8:00–9:00).

**Figure 7:**
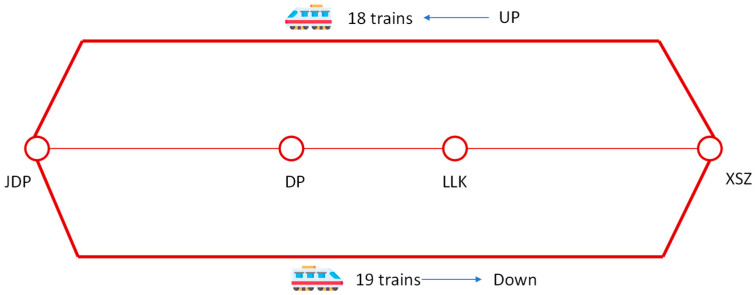
Train operation schedule for line 1 under normal operation (8:00–9:00).

The operational adjustment is as follows:

Normal Scenario: Line 1 operates a single long-turn routing (Jiandingpo [JDP]-Xiaoshizi [XSZ]), with 18 train trips in the up direction and 19 train trips in the down direction (in Figure 7).

**Figure 8:**
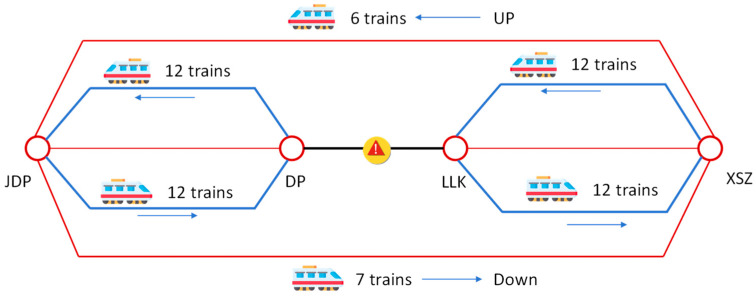
Train operation schedule for line 1 under emergency (8:00–9:00).

Disruption Scenario: A mixed routing pattern (short-turn and long-turn combined running) is adopted (as shown in Figure 8). The capacity of the long routing (JDP-XSZ) is reduced to 7 trips (down) and 6 trips (up). Meanwhile, two short routings (JDP-DP and LLK-XSZ) are added to maintain capacity in the non-disrupted sections, with 12 trips in both directions.

The simulation evaluation period is set from 7:00 to 12:00 to fully cover the processes of early passenger departure, retention/backlog accumulation, and flow dissipation.

#### 5.1.2. Candidate Path Sets and Decision Space Data

From the 35,860 OD pairs in the network, 2292 effective affected OD pairs are identified as the focus of optimization after data preprocessing. For each affected OD, a discrete alternative set is generated using the K-shortest path algorithm, comprising the Original Path and a Recommended Path (Table 3). This configuration defines a 2292-dimensional discrete solution space, where the GWO-ALNS algorithm seeks the optimal path combination to balance system efficiency and individual cost.

#### 5.1.3. Passenger Demand and Benchmarks

The passenger demand is derived from actual Automatic Fare Collection (AFC) data. During the simulation period, the total network-wide ridership is 776,707, with approximately 33,000 passengers directly affected. To evaluate the optimization performance, the “no-guidance” scenario (passengers maintain original paths) is established as the Benchmark: the total baseline travel time ($Z_{\mathrm{time}}^{0}$) is $1.137\times 10^{8}$ s, and the baseline crowding perception time ($Z_{\mathrm{crowd}}^{0}$) is $6.38\times 10^{6}$ s.

#### 5.1.4. Model Parameter and Computational Environment

The simulation incorporates operational parameters calibrated from established empirical studies. Specifically, the transfer penalty is set to $\beta_{trans}=4.41$ min [27], the maximum load factor is defined as $\eta_{max}=1.3$ [30,31], the crowding threshold is $\rho_{min}=5.0$ *p*/m^2^ [33], and the crowding time multiplier is $\alpha_{crowd}=0.1192$ [34].

Regarding the optimization algorithm, the parameters are configured as follows: the GWO population size is set to 80 with a maximum iteration number of $T_{max}=200$. The ALNS module adopts a population size of $N_{ALNS}=16$, a trigger interval of $I_{ALNS}=100$, and a starting threshold of $T_{start}=200$.

All experiments were conducted on a workstation equipped with an Intel Core i7-10,700 K CPU @ 3.80 GHz and 64 GB RAM. The simulation framework and the GWO–ALNS algorithm were implemented in Java (JDK 8) to ensure computational efficiency and reproducibility.

#### 5.1.5. Selection of Behavioral Parameters

**Figure 9:**
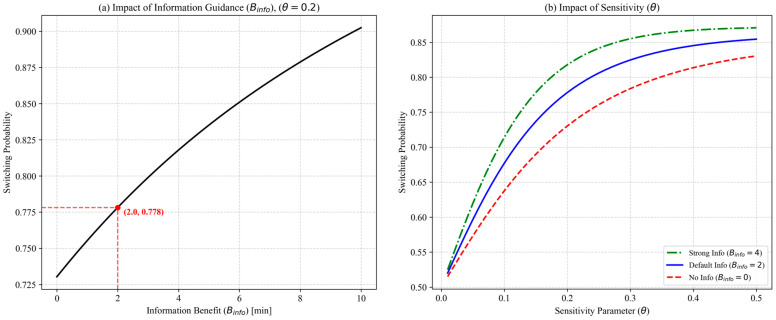
(**a**) Impact of information bias ($B_{info}$); (**b**) impact of sensitivity parameter (θ).

Although existing studies provide empirical ranges for the logit sensitivity parameter θ [28] and the information bias coefficient $B_{info}$ [29], selecting specific fixed values is necessary to ensure the numerical stability and interpretability of large-scale simulations. To calibrate these parameters considering the variability of human bias and information compliance, a sensitivity analysis was conducted on a representative OD pair (Station 0110 to Station 0318, N=300), as shown in Figure 9.

Figure 9a shows that increasing information bias $B_{info}$ raises responsiveness to guidance, but excessively large values assume uniformly high trust and near-perfect compliance, which is unrealistic during disruptions. A conservative $B_{info}=2.0$ (red marker) is chosen within the sensitive but unsaturated range (~78% switching probability). Figure 9b illustrates that, without guidance $\left(B_{info}=0\right)$, higher sensitivity θ yields little improvement, indicating that sensitivity alone cannot drive rational rerouting under uncertainty. With $B_{info}=2.0$, θ increases switching probability, but excessive values $\left(\theta >0.4\right)$ trigger overreactions to minor utility differences, inconsistent with observed passenger behavior. Accordingly, θ=0.2 (blue marker) is adopted as a prudent baseline. The resulting parameter set $\left(\theta =0.2,\;\;B_{info}=2.0\right)$ provides a conservative behavioral baseline for subsequent experiments.

### 5.2. Validation of Simulation Model Fidelity

To mitigate the high computational cost of the full-scale simulator $F_{2}$ in high-frequency iterative optimization, this study employs an incremental simulator $F_{1}$. Before applying $F_{1}$ to large-scale optimization, its fidelity and computational efficiency must be validated. To this end, a random test set of 200 guidance schemes was generated, with Induction Rates uniformly spanning 0.5–95% to cover diverse network states from partial to full intervention. For each scheme, $F_{1}$ and $F_{2}$ were run independently, and both the Fitness Value and single-simulation runtime were recorded to assess consistency and efficiency.

#### 5.2.1. Fidelity Analysis

**Figure 10:**
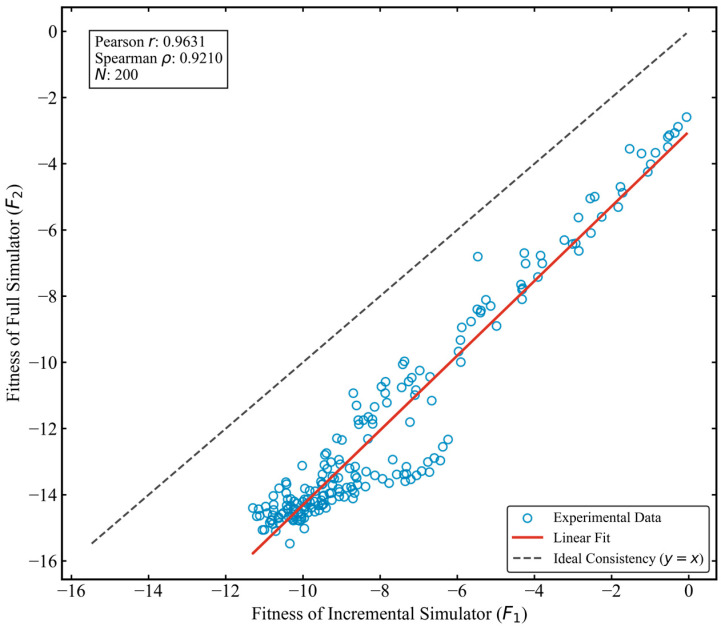
The correlation between the fitness values of $F_{1}$ and $F_{2}$.

Figure 10 illustrates the distribution of the correlation between the fitness values of $F_{1}$ and $F_{2}$. Statistical analysis reveals a high degree of consistency between the two in terms of physical mechanisms:

1. High Linear Correlation: The two exhibit a significant linear relationship, with a Pearson correlation coefficient as high as 0.9631. This indicates that although the absolute values of $F_{1}$ tend to be smaller than those of $F_{2}$ (showing a characteristic of conservative estimation) due to the simplification of certain network externalities, $F_{1}$ can accurately capture the linear variation relationship of passenger travel costs and network congestion costs as the guidance schemes change.

2. Directional Consistency & Rank Preservation: For optimization algorithms, determining “whether Solution A is better than Solution B” is more critical than accurately calculating the “specific cost of Solution A.” Experimental results show that the Spearman rank correlation coefficient between the two is 0.9210. This implies that in over 92% of cases, the quality ranking of $F_{1}$ is completely consistent with that of $F_{2}$. This extremely strong rank preservation ensures that the gradient direction provided by $F_{1}$ is correct, effectively guiding heuristic algorithms (such as GWO-ALNS) to converge toward the global optimum within the solution space.

#### 5.2.2. Efficiency Analysis

**Figure 11:**
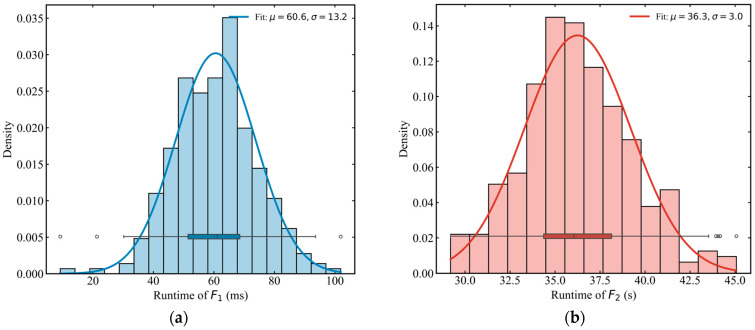
Statistical distribution of computational runtime for $F_{1}$ and $F_{2}$. (**a**) Runtime of $F_{1}$ (ms); (**b**) runtime of $F_{2}$ (s).

Figure 11 illustrates the runtime distribution of the two simulators, highlighting a stark contrast in computational performance. While a single run of the full-scale simulator $F_{2}$ requires a mean time of 36.3 s, the incremental simulator $F_{1}$ achieves the same evaluation in only 60.6 ms. This represents a radical computational acceleration of approximately 599.0 times (Mean Speedup).

This immense efficiency gain fundamentally shifts the computational paradigm for large-scale metro emergency management. For an iterative optimization task involving 1000 scheme evaluations, the total processing time is compressed from more than 10 h to approximately 1 min. This order-of-magnitude leap from “hours” to “minutes” effectively eliminates the long-standing computational bottleneck in traditional simulation-based optimization. By establishing $F_{1}$ as the high-speed core engine, the proposed framework enables massive strategy searches and dynamic decision-making to be completed within the extremely tight time windows required for real-time urban rail transit operations.

In summary, this coupled characteristic of “high rank preservation” and “high speedup ratio” enables $F_{1}$ to replace the computationally expensive $F_{2}$ in undertaking the majority of evaluation tasks during the optimization process. Therefore, subsequent sections of this paper will formally adopt $F_{1}$ as the fitness evaluation function during the iterative optimization phase, invoking $F_{2}$ only in the final scheme validation phase to achieve the optimal balance between solution accuracy and computational cost.

### 5.3. Comparative Analysis of Algorithm Performance

#### 5.3.1. Experimental Design and Evaluation Metrics

The experimental framework is systematically designed to evaluate the performance gains of the proposed hybrid architecture and the fidelity of its underlying simulation models. To establish a robust benchmark, four classic meta-heuristics—PSO, WOA, DE, and GWO—are selected as baselines, with their corresponding ALNS-hybrid variants and a Pure ALNS local search serving as control groups. This comparative setup aims to isolate the contributions of global exploration and local refinement within the hybrid framework, particularly its ability to overcome the premature convergence inherent in single-strategy heuristics.

The optimization performance is quantified across multiple dimensions, focusing on solution accuracy, robustness, and computational efficiency. Specifically, the Mean Value and Standard Deviation (Std) of fitness across independent runs are utilized to assess the upper limit of optimization capability and the stability of the solution structure, respectively. Furthermore, CPU Time is recorded to evaluate the trade-off between search precision and time cost. Crucially, to mitigate the risk of model distortion during high-speed iterations, a dual-precision shadow validation mechanism is implemented. While the incremental simulator $F_{1}$ provides rapid gradient guidance for the evolutionary process, the full-scale simulator $F_{2}$ is utilized asynchronously to verify the true cost of the optimal solutions. The synchronization between the evolutionary trajectories of $F_{1}$ and $F_{2}$ is monitored throughout the process to ensure the fidelity and reliability of the incremental guidance mechanism.

#### 5.3.2. Comparative Analysis of Experimental Results

The statistical results summarized in Table 4 assess the optimization performance of the tested algorithms under identical computational resources. In the proposed model, the fitness function is defined as a weighted reduction rate relative to the benchmark condition; therefore, a lower (more negative) fitness value indicates a greater reduction in system-wide generalized costs.

Analysis of Table 4 reveals consistent performance improvements across all baseline algorithms following the integration of the ALNS operator, with enhancement magnitudes varying between 12.0% and 30.2%. Among these, WOA-ALNS achieves the largest relative improvement (+30.2%), confirming the “Destroy–Repair” mechanism’s capacity to offset WOA’s weak search behavior in discrete spaces. However, the GWO-ALNS framework attains the best overall fitness value (−18.03), corresponding to an 18% reduction in weighted generalized cost compared with the unguided benchmark. This superior performance reflects the synergy between GWO’s efficient exploitation and ALNS’s strong ability to escape local extrema. Furthermore, the competitive outcome of Pure ALNS (−16.30)—surpassing baseline WOA and PSO—highlights the efficiency of domain-specific adaptive operators over general random search in identifying high-quality solutions within complex rail networks.

**Figure 12:**
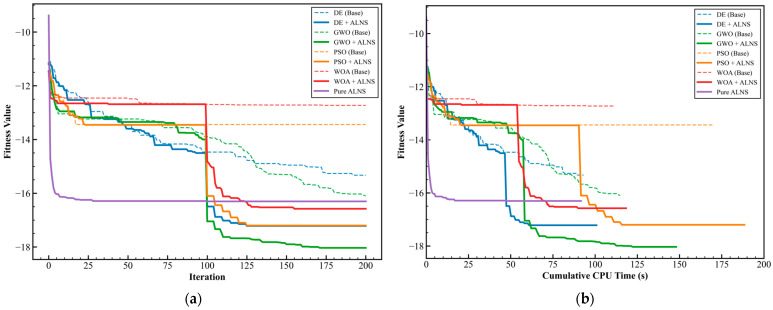
Comparison of convergence curves of different algorithms. (**a**) Fitness vs. iteration. (**b**) Fitness vs. CPU time.

The convergence profiles in Figure 12a (iteration domain) and Figure 12b (CPU time domain) further illustrate the search dynamics. Standard PSO and WOA exhibit premature convergence, flattening their fitness curves between generations 50 and 80 due to limited population diversity in the 2292-dimensional search space. In contrast, the activation of ALNS around generation 100 produces a distinctive staircase-shaped descent, where each abrupt drop reflects a successful ALNS intervention that refines the global best solution. Although Pure ALNS converges rapidly, it becomes trapped in local optima by approximately the 50th generation. The hybrid GWO-ALNS strategy, however, combines GWO’s broad early-stage exploration with ALNS’s precise late-stage refinement, demonstrating the most consistent convergence toward the global optimum under comparable computational budgets.

#### 5.3.3. Validation of Directional Consistency in Dynamic Optimization

**Figure 13:**
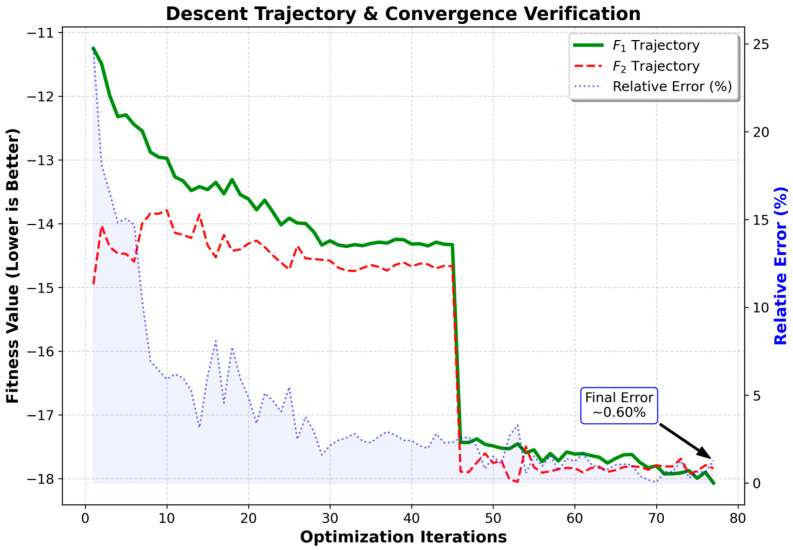
Fitness value trajectories of $F_{1}$ and $F_{2}$ during dynamic optimization.

To further confirm the reliability of establishing the incremental model ($F_{1}$) as the core solver, improved solutions obtained during the solution process of the GWO-ALNS algorithm were extracted. Fitness values were calculated separately based on $F_{1}$ and $F_{2}$ solvers to deeply analyze the gradient guidance capability and terminal approximation accuracy of the two during dynamic optimization. The analysis results are shown in Figure 13.

(1) Gradient Directional Locking in Dynamic Optimization

Experiments show that $F_{1}$ possesses extremely high dynamic sensitivity to physical field changes in the full simulation ($F_{2}$), especially during the critical stage where the algorithm escapes from local optima (e.g., Generation 45), exhibiting a “cliff-like” synchronous response highly consistent with the true value. Despite minor perturbations during the stable period, $F_{1}$ maintains strict directional locking on the Dominant Gradient that determines the search path.

(2) Adaptive High Fidelity in Convergence

As the solution structure tends towards stability, $F_{1}$ demonstrates excellent adaptive approximation capability. Its relative error with $F_{2}$ significantly converges to 0.60% in the final 10 generations. This validates the high degree of agreement of the incremental simulation with non-linear physical laws under the final equilibrium state of the network, guaranteeing the credibility of the final evaluation results and justifying its effectiveness in replacing the expensive full simulation for large-scale iterative optimization.

### 5.4. Result Analysis and Visualization

Based on the global optimal guidance scheme calculated by the GWO-ALNS algorithm, the Full Simulator was used to deduce the network state before and after guidance, obtaining precise passenger travel time and train-section load factor data. This section presents a comparative analysis from three dimensions: microscopic individual benefit, macroscopic network state, and section flow evolution.

#### 5.4.1. Efficacy Evaluation of the Optimal Guidance Scheme

(1) Time Efficiency Analysis

OD Granularity Analysis: Among the 2292 OD pairs affected by the sudden incident across the network, the algorithm successfully implemented effective guidance for 1371 OD pairs (accounting for 57.5%).

Benefited Type: 1271 OD pairs (55.5%) saw a significant decrease in travel time, directly benefiting from better paths.

Altruistic Type: 21 OD pairs (0.9%) accepted detour paths with slightly increased time. This “sacrifice” is crucial for relieving core bottleneck pressure, reflecting the local compromise of individual optimality for system optimality.

Neutral Type: 25 OD pairs had unchanged time; the remaining 975 OD pairs (42.5%) remained unchanged because their original paths were still currently optimal.

Passenger Dimension Analysis: Among the 33,000 affected passengers, 14,896 actually executed route-changing behavior (information response rate was 87.3%). After guidance, the average travel time of the affected group dropped from 57.42 min to 50.26 min, an average saving of 7.16 min per person (a decrease of 12.5%), cumulatively saving about 3940 h of total social time.

(2) Congestion Relief Efficacy Analysis

Perceived Cost Dimension: The total congestion perception penalty value of the entire network dropped from 6.38 × 10^6^ s to 4.88 × 10^6^ s, a decrease of 23.5%, indicating that the guidance scheme significantly improved ride comfort while enhancing travel speed.

Physical Load Dimension: Table 5 shows the distribution changes of train-section load factors. After implementing guidance, the number of sections in a “severely congested” state (load factor > 1.1) decreased by 28 (a decrease of 36%), and “congested” sections (0.8–1.1) decreased by 7. This confirms that the algorithm has significant “Peak Shaving” capability, effectively guiding passenger flow from oversaturated areas to non-saturated areas.

#### 5.4.2. Spatiotemporal Evolution Analysis of Network Section Flow

**Figure 14:**
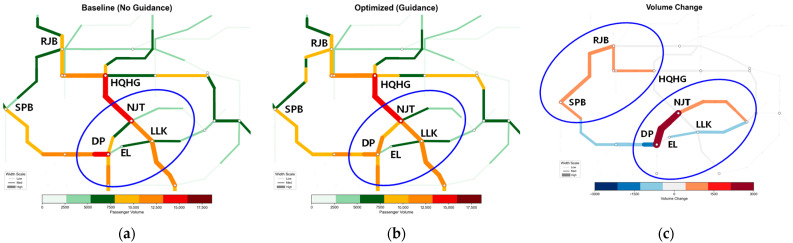
Spatiotemporal heat distribution of network section flow (8:00−8:30). (**a**) Baseline (No guidance); (**b**) optimized (guidance); (**c**) volume change.

**Figure 15:**
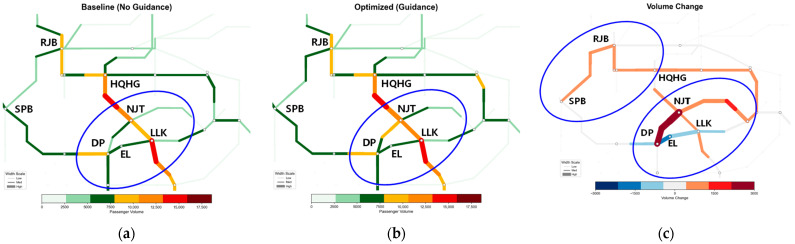
Spatiotemporal heat distribution of network section flow (8:30–9:00). (**a**) Baseline (No guidance); (**b**) optimized (guidance); (**c**) volume change.

**Figure 16:**
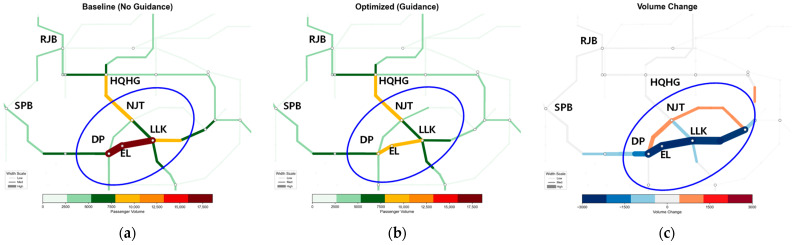
Spatiotemporal heat distribution of network section flow (9:00–9:30). (**a**) Baseline (No guidance); (**b**) optimized (guidance); (**c**) volume change.

**Figure 17:**
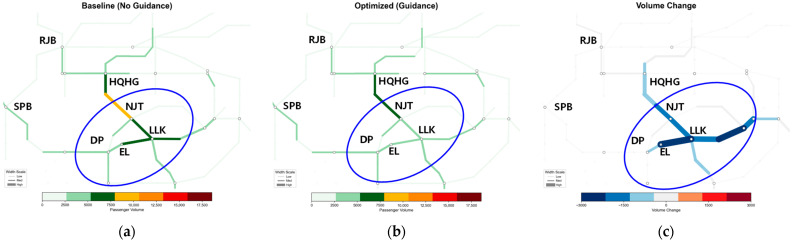
Spatiotemporal heat distribution of network section flow (9:30–10:00). (**a**) Baseline (No guidance); (**b**) optimized (guidance); (**c**) volume change.

Figure 14, Figure 15, Figure 16 and Figure 17 illustrate the spatiotemporal heat distribution of network section flows between 08:00 and 10:00. To facilitate comparative analysis, critical regions exhibiting significant flow variations are marked with blue ellipses. In the difference maps (sub-figure c), red lines (positive values) indicate flow increments on detour paths, while blue lines (negative values) denote flow reductions in congested sections.

Based on these visualizations, the regulatory mechanism of the guidance strategy is revealed across the disruption lifecycle:

(1) Congestion Outbreak Period (08:00–08:30)—Figure 14

During the initial disruption, the algorithm rapidly activates a hierarchical diversion mechanism, as reflected in the flow difference patterns in Figure 14c. Thick red segments emerge along the proximal detour path DP–NJT, indicating that a large volume of passengers are diverted away from the bottleneck. Correspondingly, the faulty section DP–LLK is dominated by blue segments, showing a substantial flow reduction relative to the baseline. At the same time, light red increments appear on the upstream section SPB–RJB, confirming pre-emptive interception of demand via the outer ring line and effective bypass of the core congestion zone.

(2) Sustained Control Period (08:30–09:00)—Figure 15

As shown in Figure 15c, the redistribution pattern stabilizes. The proximal detour path DP–NJT consistently exhibits thick red segments, functioning as a stable bypass channel. In contrast, the faulty section DP–LLK remains dominated by blue segments, indicating continuous flow suppression. This effectively limits passenger accumulation at stations and prevents congestion spillback to adjacent lines.

(3) Dissipation and Recovery Period (09:00–10:00)—Figure 16 and Figure 17

As demand subsides, the strategy shifts from diversion to accelerating queue dissipation. During 09:00–09:30, Figure 16c shows pronounced blue segments along the bottleneck section, indicating rapid clearance of residual congestion, whereas the baseline suffers from a persistent long-tail effect. Meanwhile, orange segments in the upstream area indicate that distal diversions remain active to prevent rebound. By 09:30–10:00, as shown in Figure 17c, the colored difference segments across both zones thin and fade, signaling the deactivation of guidance. The sustained blue pattern on the faulty section confirms that congestion hysteresis is eliminated, enabling network recovery approximately 30 min earlier than the baseline.

#### 5.4.3. Spatiotemporal Analysis of Train Load Relief on Line 1

**Figure 18:**
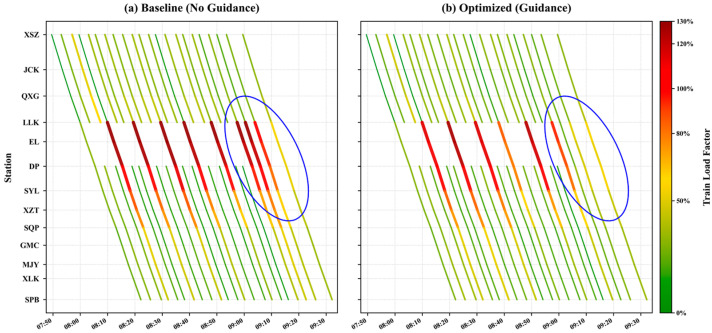
Spatiotemporal trajectory heatmap of downward train load factor on line 1.

**Figure 19:**
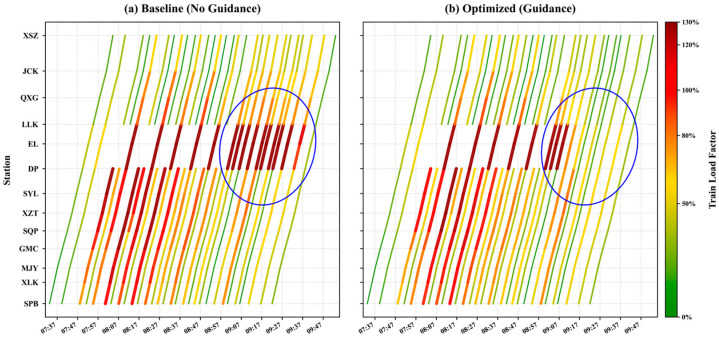
Spatiotemporal trajectory heatmap of upward train load factor on line 1.

Figure 18 and Figure 19 compare the spatiotemporal evolution of train load factors on the critical Line 1 corridor (SPB–XSZ) under baseline and optimized guidance scenarios. The most prominent differences, highlighted by the circled regions, clearly illustrate how the proposed strategy accelerates congestion dissipation and network recovery.

In the downward direction (Figure 18), the circled area highlights a contrast during the recovery phase. In the baseline scenario (Figure 18a), section capacity reduction results in sustained high-load train sections (red segments), with three consecutive sections affected. In the optimized scenario (Figure 18b), congestion is limited to one high-load section, indicating that the guidance strategy mitigates high-load propagation and allows the corridor to return to unsaturated conditions earlier.

The improvement is even more pronounced in the upward direction (Figure 19). In the baseline scenario (Figure 19a), approximately ten train sections remain heavily loaded in the circled region. After guidance (Figure 19b), the red zone is compressed to three sections, corresponding to a reduction of 70% in sustained high-load conditions, demonstrating that the strategy effectively alleviates congestion and enhances corridor resilience under asymmetric passenger flow conditions.

## 6. Discussion

### 6.1. Linear Assumption Under Extreme Congestion

A critical question is whether the linear superposition assumption of the incremental model $\left(F_{1}\right)$ remains valid under extreme congestion, characterized by severe queueing and passenger accumulation. Passenger demand was scaled from 1.25× to 2.0×, covering scenarios from heavy load to extreme overcrowding, to evaluate the model’s performance under these conditions.

**Figure 20:**
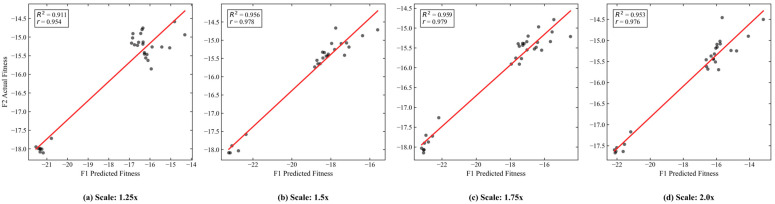
Correlation between fitness values of $F_{1}$ and $F_{2}$ under extreme congestion.

Figure 20 shows that, despite systematic deviations in absolute fitness values between $F_{1}$ and $F_{2}$, the coefficient of determination $\left(R^{2}\right)$ remains consistently high (0.91–0.95) across all demand scales, indicating that the linear component of capacity consumption continues to dominate the objective function.

**Figure 21:**
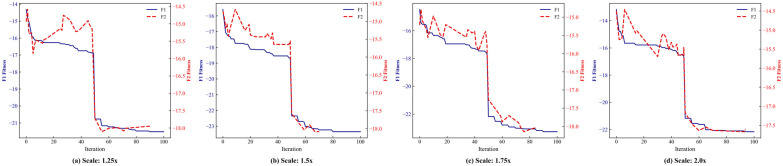
Fitness value trajectories of $F_{1}$ and $F_{2}$ under extreme congestion.

Dynamic trajectory analysis (Figure 21) further demonstrates that, although absolute values differ slightly, the evolutionary trends of $F_{1}$ and $F_{2}$ remain highly synchronized. During the ALNS, whenever a solution identified by $F_{1}$ improves fitness, $F_{2}$ exhibits a corresponding synchronized descent, confirming that $F_{1}$ accurately captures the global optimization gradient.

These results indicate that, while absolute estimation errors exist under extreme congestion, $F_{1}$ reliably preserves rank fidelity and directional guidance, validating its use as an efficient surrogate within the proposed hierarchical framework.

### 6.2. Sensitivity and Robustness Analysis of Weight Coefficients

Since the proposed optimization framework is embedded within an iterative simulation environment, a deterministic decision mechanism is required at each step. While Pareto optimization theoretically offers a comprehensive set of non-dominated solutions, it creates a practical dilemma: the simulation model requires a unique, specific input to calculate the next state of crowd dynamics. A set of multiple Pareto solutions cannot simultaneously drive a single simulation run.

To resolve this, we employ the Weighted Sum Method to scalarize the comprehensive optimization problem into a single fitness value, ensuring a clear evolutionary direction for the algorithm. Consequently, to address the potential subjectivity inherent in weight assignment, this section conducts a robust sensitivity analysis on the weight coefficients (α, β, γ). We analyze the trade-offs among travel efficiency, congestion levels, and guidance costs to verify that the chosen weights lead to stable and effective system performance.

#### 6.2.1. Sensitivity Analysis of Time and Congestion Weights

**Figure 22:**
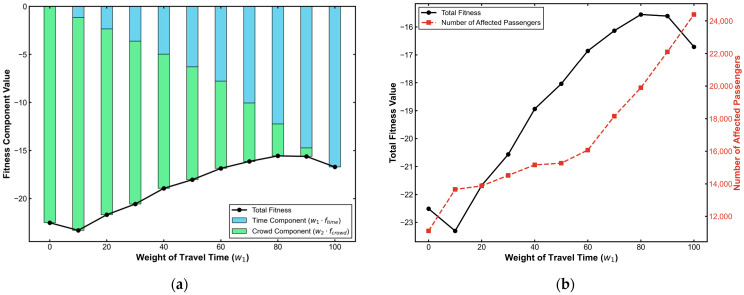
Sensitivity analysis of time weight ($w_{1}$). (**a**) Fitness component value; (**b**) total fitness value and affected passenger numbers.

Under the condition $w_{3}=0$ and $w_{1}+w_{2}=100$, varying $w_{1}$ simulates the strategic shift from congestion-oriented to speed-oriented preferences. The results, shown in Figure 22, reveal a pronounced non-linear response in system performance.

First, the global optimum exhibits a distinct “left-skewed” distribution. The optimal fitness occurs at $w_{1}=10$ rather than the equilibrium point ($w_{1}=50$). This implies that under severe capacity constraints, prioritizing congestion relief generates higher positive externalities than purely pursuing speed, thereby maximizing network-wide efficiency.

Second, a threshold effect exists between intervention scale and marginal utility. While the number of guided passengers increases monotonically with $w_{1}$, fitness gains diminish sharply beyond $w_{1}>60$. At this stage, the marginal benefit of additional rerouting is outweighed by system perturbation costs, confirming that effective route guidance has a physical limit defined by network capacity.

#### 6.2.2. Sensitivity Analysis of Rerouting Cost Weight

**Figure 23:**
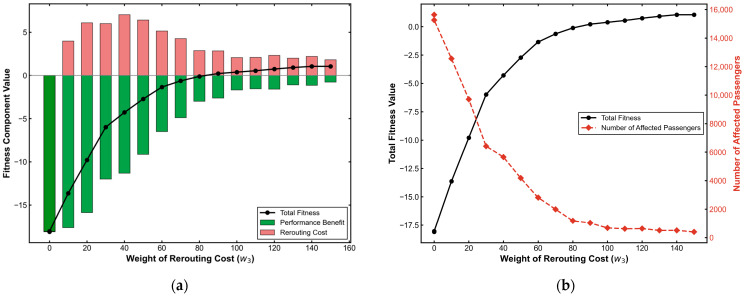
Sensitivity analysis of rerouting cost weight ($w_{3}$). (**a**) Fitness component value; (**b**) total fitness value and affected passenger numbers.

A rerouting penalty $w_{3}$ is introduced (with fixed $w_{1}$ = 50, $w_{2}$ = 50) to investigate the screening mechanism for high-value guidance targets. Results are shown in Figure 23.

The $w_{3}$ parameter functions as a “High-pass Filter” for sparsification. The guided population shows an L-shaped decay as $w_{3}$ increases, stabilizing after an “Elbow Point” at $w_{3}=30$. This mechanism automatically filters out 59% of inefficient detours (low-impact passengers), effectively isolating the core group that contributes most to system relief. Furthermore, the analysis identifies the economic boundary of the strategy. The fitness curve reflects a Break-Even Point at $w_{3}=83$. Beyond this threshold, the generalized cost of rerouting exceeds its benefits.

### 6.3. Comparative Assessment Against Standard Engineering Strategies

To assess the practical relevance of the proposed GWO-ALNS framework, it was compared against a Dynamic K-Shortest Path (DKSP) strategy [35], a common route guidance logic in Advanced Traveler Information Systems (ATISs). DKSP provides travelers with a limited set of alternative routes and probabilistically assigns choices based on real-time travel costs. For a fair comparison with the binary switching decisions in our model, the benchmark was restricted to K=2, allowing passengers to choose between the original path and the best alternative via a Logit formulation.

The two methods exhibit markedly different system behaviors. DKSP converges to an average fitness of −13.37, reflecting a user-equilibrium-like state driven by myopic individual decisions. While it offers some congestion relief compared to no guidance, it cannot fully resolve complex bottleneck interactions in tightly coupled metro networks. In contrast, GWO-ALNS achieves a fitness of −18.03, representing a 34.8% improvement and demonstrating that dynamic route information alone is insufficient, while intelligent system-level coordination is essential to approach the true network optimum.

## 7. Conclusions

Addressing the challenges of real-time responsiveness and precision in passenger flow control under sudden urban rail disruptions, this paper proposes a dual-layer simulation-driven GWO-ALNS hybrid intelligent optimization framework. This research provides not only algorithmic support for the autonomous emergency response of fully automated lines but also a new quantitative paradigm for the resilience management of large-scale networks. The principal conclusions and contributions are summarized as follows:

Formulating a Comprehensive Route Guidance Optimization Model: A comprehensive integer programming model is set up to simultaneously consider travel time, congestion perception time, and information costs. To capture passenger behavior under disruptions, a Logit choice model with information bias is embedded, and sensitivity analyses are conducted to determine reasonable parameter settings, reflecting passengers’ varying acceptance of guidance information and supporting system-level optimization during disruptive events.

Developing a Bi-Level Simulation Evaluation Mechanism: A bi-level simulation framework is employed to precisely assess passenger flow dynamics in disrupted metro networks. The incremental simulator $F_{1}$, based on differential computation, achieves a 599-fold speedup while maintaining high fidelity with full-scale simulations (Pearson correlation > 0.96). Robustness tests show that even under extreme congestion (2× demand saturation), $F_{1}$ preserves high rank fidelity $\left(R^{2}>0.91\right)$, confirming the feasibility of simulation-driven optimization within the “golden decision window” of emergency response.

Designing a Hybrid GWO-ALNS Framework: The hybrid GWO-ALNS algorithm combines global search with local refinement and embeds domain-specific operators, including “Congestion Bottleneck Removal.” Its domain knowledge-based ALNS leverages OD-level diagnostics to guide efficient and precise local optimization of critical OD pairs. Compared to baseline optimization algorithms, it improves solution quality by 12–30%, and against a Dynamic K-Shortest Path (DKSP) benchmark, it achieves a 34.8% improvement in fitness, enabling simultaneous optimization at both individual and system levels.

Demonstrating the Practical Value of the Guidance Framework: Empirical results from the Chongqing Rail Transit case show that coordinating passenger flows among affected OD pairs reduces severely congested sections by 36% and improves average travel efficiency by 12.5% (7.16 min). These results confirm the practical applicability of the proposed guidance framework, demonstrating its effectiveness in inducing targeted passenger rerouting and enhancing system-level performance under disruptions.

Future study plans include (i) advancing the optimization framework to multi-objective evolutionary optimization to generate Pareto-optimal solution sets; (ii) improving the incremental simulation model by incorporating non-linear corrections for spatiotemporal path redistribution of stranded passengers in locally oversaturated segments, thereby mitigating approximation bias induced by linear assumptions and enhancing result robustness; (iii) extending the framework to a multimodal super-network (e.g., bus bridging, shared mobility) by leveraging external sensing data to coordinate cross-system passenger guidance; and (iv) accelerating the full-scale simulator $F_{2}$ via parallel or hybrid mesoscopic methods to support real-time initialization and validation in ultra-large-scale networks.

## Figures and Tables

**Table 1:** Example of route reconstruction for typical affected OD pairs.

Original OD	Optional Path (r)	Path Routing	Decision Characteristics
A → D	r=0 (Original Path)	A → B (Disruption/Wait) → C → D	Passive Waiting: Requires waiting at Station A for recovery.
	r=1 (Detour)	A → E → D	Active Detour: Utilizes transfer at Station E; incurs additional transfer costs.
	r=2 (Feeder)	A → B → Bus → D	Road Feeder: Utilizes ground buses; significantly affected by traffic conditions.

**Table 2:** Table of model symbol definitions.

Symbol	Definition
Sets & Indices	
K	Set of affected OD pairs, indexed by k
$P_{k}$	Set of directly affected passengers belonging to OD pair k, indexed by p.
$R_{k}$	Set of available path schemes for OD pair k, indexed by r (r = 0: original path; r = 1: recommended path).
N	Set of stations (nodes) in the network, indexed by n.
V	Set of trains actually operating in the network, indexed by v.
$E_{v}$	Set of sections in the operation trajectory of train v, indexed by e.
Parameters	
$N_{k}$	Total number of affected passengers for OD pair k.
$N_{total}$	Total number of affected passengers in the network ($\sum N_{k}$).
$Z_{time}^{0}$	Baseline total physical travel time (no-guidance scenario), used for normalization.
$Z_{crowd}^{0}$	Baseline total congestion perception penalty (no-guidance scenario), used for normalization.
$C_{v}$	Maximum passenger carrying capacity of train v.
$t_{v,e}$	Physical running time of train v on section e (excluding dwell time).
$\alpha_{crowd}$	Crowding multiplier reflecting the magnified perception of time in high-density environments.
$\beta_{trans}$	Equivalent time penalty for a single transfer operation (s).
$B_{info}$	Information compliance bias utility, characterizing passenger trust in official recommendations.
θ	Sensitivity coefficient of the Logit path choice model.
$w_{1},w_{2},w_{3}$	Weight coefficients for efficiency, experience, and information cost objectives, respectively.
Variables	
X	Global guidance strategy vector composed of binary variables for all OD pairs.
$x_{k}$	Binary decision variable: equal to 1 if guidance information is sent to OD pair k; 0 otherwise.
$y_{p,r}$	Path choice state variable: equal to 1 if passenger p finally chooses path r; otherwise, it is 0.
$Q_{v,e}$	Actual passenger load of train v on section e during simulation.
$\rho_{v,e}$	Standing density of train v on section e (*p*/m^2^).

**Table 3:** Representative path samples for different disruption scenarios.

Disruption Type	OD Pair	Original Path (Route/Cost)	Recommended Path (Route/Cost)	Δ Cost
Origin Blocked	EL → XSZ	01-UP-0106-0102/725 s	01-UP-0105-0102/1470 s	+102.8%
Dest. Blocked	JDP → EL	01-UP-0124-0106/2555 s	01-UP-0124-0107/3615 s	+41.5%
Link Blocked	DP → JCK	01-UP-0107-0103/665 s	02-UP-0209-0201/1030 s	+54.9%

**Table 4:** Statistical comparison of optimization performance and computational cost.

Algorithm	Variant	Best Fitness	Improvement	Avg. Runtime (s)
GWO	Base	−16.10	-	114.8
	GWO-ALNS	−18.03	+12.0%	148.0
DE	Base	−15.33	-	93.2
	DE-ALNS	−17.22	+12.3%	100.8
PSO	Base	−13.44	-	170.2
	PSO-ALNS	−17.20	+28.0%	188.5
WOA	Base	−12.73	-	111.3
	WOA-ALNS	−16.58	+30.2%	118.4
Pure ALNS	-	−16.30	-	91.7

**Table 5:** Comparison of network section load factor distribution before and after guidance.

Load Factor Range	Congestion Level	Before Guidance	After Guidance	Change in Count
0–0.8	Comfortable	2933 sections	2968 sections	+31
0.8–1.1	Congested	110 sections	103 sections	−7
1.1–1.3	Severely Congested	78 sections	50 sections	−28

## Data Availability

Data are only available upon request due to restrictions regarding, e.g., privacy and ethics. The data presented in this study are available from the corresponding author upon request. The data are not publicly available due to their relation to other ongoing research.
